# Supplementary figures and images for: Optimization of T-DNA architecture for Cas9-mediated mutagenesis in Arabidopsis
Source: PLoS One. 2019 Jan 9;14(1):e0204778. doi: 10.1371/journal.pone.0204778 (PMC6326418; doi:10.1371/journal.pone.0204778)

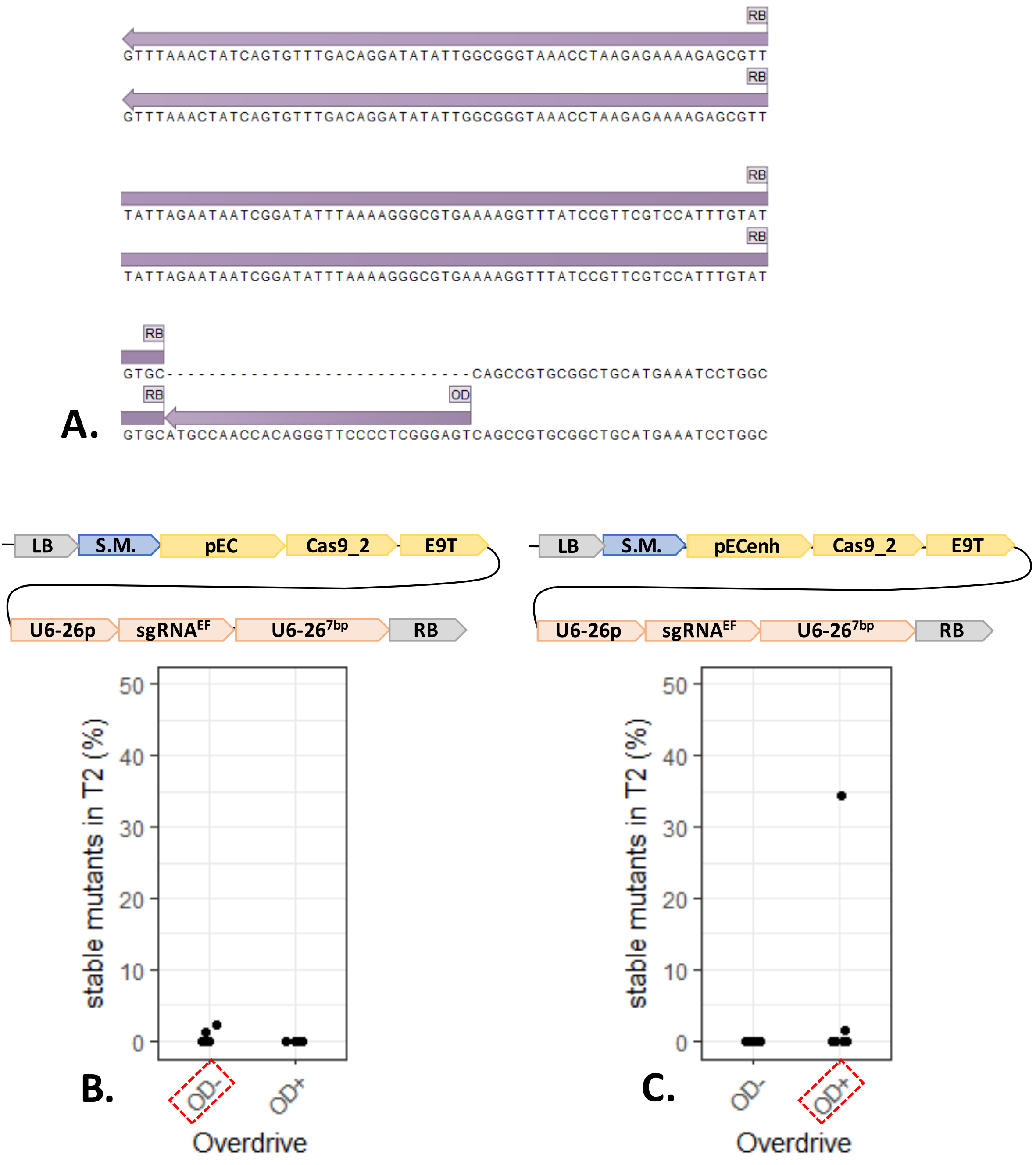

Supplement: S1 Fig — A. Sequence of the right border with (pICSL4723) or without (pAGM4723) the overdrive sequence. B. and C. Each panel represents a vector comparison in the same context. Vectors can be compared within each panel, not from one panel to another. The modules have been assembled by Golden Gate into pICSL4723 (OD+, with an overdrive) or pAGM4723 (OD-, without an overdrive) and transformed into Col-0 via Agrobacterium tumefaciens strain GV3101. LB: Left Border. SM: Sel. Marker (Glufosinate resistance gene). EC1.2: 1014 bp of the At2g21740 promoter. EC_enh.: 752 bp of the At2g21740 promoter fused to 548 bp of the At1g76750 promoter. Cas9_2: Fauser et al., 2014 [13]. E9T: 631 bp of the Pisum sativum rbcS E9 terminator. U6-26p: 205 bp of the At3g13855 promoter. sgRNAEF: “extension-flip” sgRNA. U6-26T: 7 bp of the At3g13855 terminator. RB: Right Border. The sgRNA targets ADH1. CRISPR activity measured in % of homozygous or biallelic stable mutants in the second generation after transformation (T2). Each dot represents an independent T2 family. Bold and underlined: Most active construct(s) for each panel. The overdrive sequence can increase the integration efficiency [21]. In one comparison the presence of the overdrive results in slightly better activity (C), but in another one it did not (B). We concluded that the presence of an overdrive does not influence the CRISPR efficiency. Thus, we could compare constructs independently of the presence of an overdrive. (TIFF) [file pone.0204778.s006.tiff]

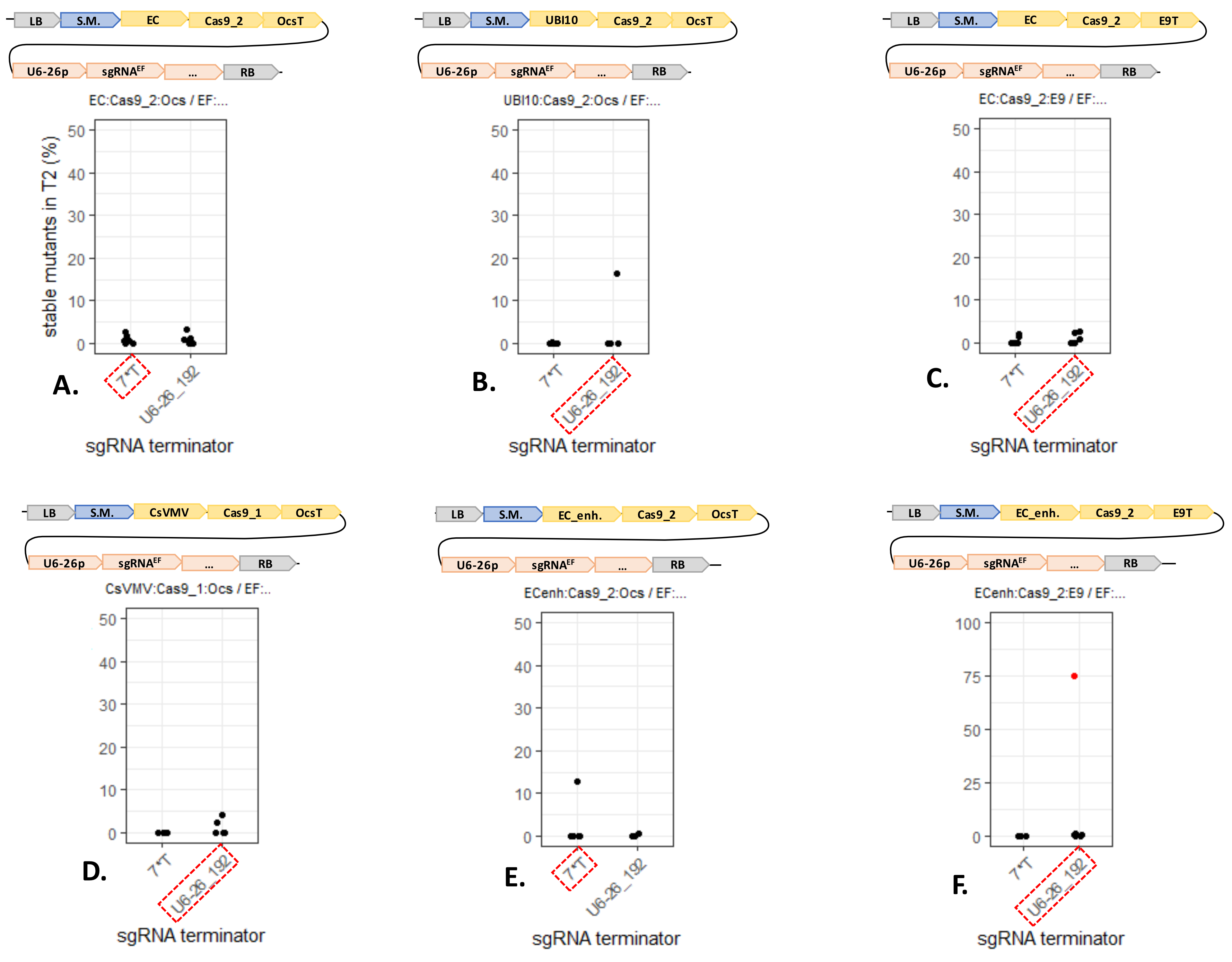

Supplement: S2 Fig — A. to F. Each panel represents a terminator comparison in the same context. Terminators can be compared within each panel, not from one panel to another. The modules were assembled into pICSL4723 (RB+OD, with an overdrive) or pAGM4723 (RB, without an overdrive) and transformed into Col-0 via Agrobacterium tumefaciens strain GV3101. LB: Left Border. SM: Sel. Marker (Glufosinate resistance gene). CsVMV: 517 bp of a promoter from Cassava Vein Mosaic virus. UBI10: 1327 bp of the At4g05320 promoter. EC1.2: 1014 bp of the At2g21740 promoter. EC_enh.: 752 bp of the At2g21740 promoter fused to 548 bp of the At1g76750 promoter. Cas9_1: Mali et al., 2013 [3]. Cas9_2: Fauser et al., 2014 [13]. E9T: 631 bp of the Pisum sativum rbcS E9 terminator. OcsT: 714 bp of the Agrobacterium tumefaciens octopine synthase terminator. EF: 205 bp of the At3g13855 promoter controlling the expression of an “extension-flip” sgRNA. U6-26T: 7 or 192 bp of the At3g13855 terminator. B. Five lines were tested for 7*T instead of six. The sgRNA targets ADH1. CRISPR activity measured in % of homozygous or biallelic stable mutants in the second generation after transformation (T2). Each dot represents an independent T2 family. Red dot: All the T2 lines from this family carry the same mutation, indicating a mutation more likely inherited from the T1 parent rather than being de novo from the T2 line. Bold and underlined: Most active construct(s) for each panel. (TIFF) [file pone.0204778.s007.tiff]
